# Cleavage of the N≡N Triple Bond and Unpredicted Formation of the Cyclic 1,3‐Diaza‐2,4‐Diborete (FB)_2_N_2_ from N_2_ and Fluoroborylene BF

**DOI:** 10.1002/anie.202106984

**Published:** 2021-06-26

**Authors:** Bing Xu, Helmut Beckers, Haoyu Ye, Yan Lu, Juanjuan Cheng, Xuefeng Wang, Sebastian Riedel

**Affiliations:** ^1^ School of Chemical Science and Engineering Department Shanghai Key lab of Chemical Assessment and Sustainability Tongji University Shanghai 200092 China; ^2^ Institut für Chemie und Biochemie – Anorganische Chemie Freie Universität Berlin Fabeckstrasse 34–36 14195 Berlin Germany

**Keywords:** aromaticity, dinitrogen activation, fluoroborylene, puckered B_2_N_2_ ring

## Abstract

A complete cleavage of the triple bond of N_2_ by fluoroborylene (:BF) was achieved in a low‐temperature N_2_ matrix by the formation of the four‐membered heterocycle FB(*μ*‐N)_2_BF, which lacks a *trans*‐annular N−N bond. Additionally, the linear complex FB=N−N=BF and cyclic FB(*η* ^2^‐N_2_) were formed. These novel species were characterized by their matrix infrared spectra and quantum‐chemical calculations. The puckered four‐membered‐ring B_2_N_2_ complex shows a delocalized aromatic two‐electron π‐system in conjugation with the *exo*‐cyclic fluorine π lone pairs. This work may contribute to a rational design of catalysts based on borylene for artificial dinitrogen activation.

## Graphical abstract

The four‐membered heterocycle FB(*μ*‐N)_2_BF is obtained from fluoroborylene, FB, and N_2_. It shows a complete cleavage of the N_2_ triple bond and a delocalized aromatic two‐electron π‐bond conjugated with the *exo*‐cyclic fluorine π lone pairs, indicating the importance of fluorine‐specific interactions.

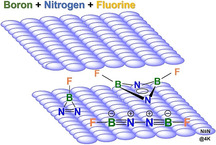

## Introduction

**Scheme 1:**
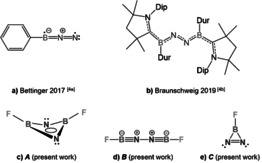
Five different binding modes of N_2_ to borylenes.

The cleavage of the N≡N triple bond (one of the strongest chemical bond) is a long‐standing task in chemistry.^[1]^ Since the discovery of the Haber‐Bosch process for producing ammonia from H_2_/N_2_ in the first decade of the 20^th^ century, plenty of transition‐metal (TM) complexes have been discovered to activate and functionalize thermodynamically stable and kinetically inert dinitrogen (N_2_) under more ambient conditions.^[2]^ For *p*‐block elements the examples of N_2_ binding are mainly contributed from boron compounds such as borylenes (:BR).^[3]^ The boron atom in borylene possess both, a lone pair of electrons (HOMO) and an energetically low‐lying empty *p* valence orbital (LUMO). Borylenes are therefore excellent candidate for mimicing transition‐metal reactivity.^[4]^ The reaction of N_2_ with free phenylborylene, :BPh, under matrix conditions has been shown to yield the linear adduct PhB=NN in a triplet ground state, underscoring the use of borylenes as candidates for N_2_ binding (Scheme 1 a).^[4e]^ A boron‐based fixation of N_2_ has very recently also been reported using a phenylborylene stabilized by a bulky carbene ligand, [(CAAC)DurB] (CAAC=cyclic alkylamino carbene, Dur=2,3,5,6‐tetramethylphenyl), leading to the end‐on bridging complex [{(CAAC)‐DurB}_2_(*μ* ^2^‐N_2_)]^[4b]^ in which the N−N bond length [1.248(4) Å] lies in the range of N=N double bonds (Scheme 1 b).^[5]^

The reaction of laser‐ablated boron atoms with N_2_ molecules upon co‐deposition onto a cooled (4 K) CsI window has previously been studied.^[6]^ These studies provided a variety of mono and diboron nitrogen compounds of the type BN_2_, B_2_N, and B_2_N_2_ as well as linear NNBN.^[6]^ It has recently been shown, that addition of CO to the boron‐N_2_ deposits gives rise to CO complexes of the BN_2_ isomers, such as the chain‐molecules NNBCO and NBNCO, cyclic (*η* ^2^‐N_2_)BCO, and the diisocyanat B(NCO)_2_.^[7]^

## Results and Discussion

Here we report on novel fluoroborylene (:BF) : N_2_ compounds, the cyclic diaza‐diborete FB(*μ*‐N)_2_BF(***A***), its linear FB=N−N=BF isomer (***B***), and cyclic fluorodiazaboririne, FB(*η* ^2^‐N_2_) (***C***) which was previously predicted^[8]^ (Scheme 1, c–e). They are selectively formed upon co‐deposition of laser‐ablated boron atoms with elemental fluorine in an N_2_ gas stream at cryogenic temperatures (4±1 K, for experimental details see the Supporting Information). Here, dinitrogen molecules act as both, reactants and host matrix. The cyclic compounds ***A*** and ***C*** are of aromatic nature due to the presence of a delocalized 2π electron bond which is in conjugation with the π lone pairs of the *exo*‐cyclic F atoms indicating a type of fluorine specific interactions.

**Figure 1:**
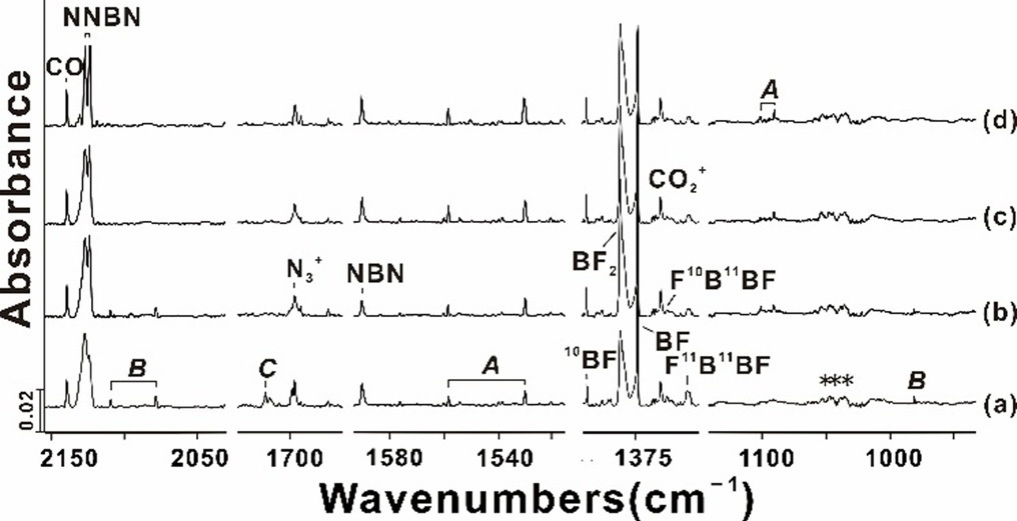
Excerpts from the IR spectrum obtained using a natural boron target with 0.5 % F_2_ in N_2_ matrix: **(a)** co‐deposition of B+0.5 % F_2_ for 120 min, **(b)** after annealing to 15 K, **(c)** after subsequent *λ*=273 nm irradiation for 30 min, and **(d)** further annealing to 15 K. Unknown species are indicated by asterisks.

Figure 1 shows infrared spectra obtained after laser‐ablated natural boron atoms co‐deposited with a 0.5 % F_2_/N_2_ mixture in a 4 K dinitrogen matrix. The reaction products induced by annealing and photolysis are indicated. In addition to the three novel fluoroborylene : N_2_ products ***A***–***C*** binary boron fluorides BF_*n*_ (*n*=1–3) and the molecular boron nitrides NBN and NNBN^[6]^ were obtained in the present study, while the previously reported diboron compounds BBNN and BNBN^[6]^ were barely observed (Figure 1 and Table S1). Although BF_2_ and BF_3_ are also produced,^[9]^ the reaction conditions were optimized to achieve a maximum yield of fluoroborylene, BF (see experimental details in the Supporting Information). The strong bands for ^11^BF and ^10^BF were found at 1370.6 and 1412.4 cm^−1^, respectively (Figure 1, Table S2). These optimized conditions enabled us to tentatively assign three weak bands to different ^10/11^B isotopologues of difluorodiborene, FBBF, at 1327.3 (^11^B^11^B), 1348.5 (^10^B^11^B) and 1370.3 cm^−1^ (^10^B^10^B) in experiments using a natural boron target in N_2_ and ^15^N_2_ matrices (Figure 1 and S3). Owing to its high reactivity, experimental spectra of free FBBF have not yet been reported, although complexes of FBBF with electron‐rich transition metals have been investigated.^[10]^

**Figure 2:**
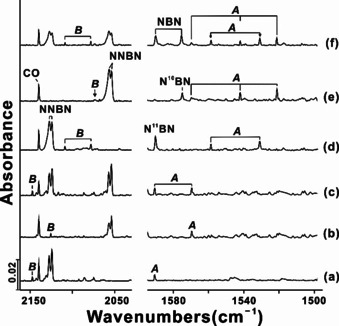
Excerpts from the IR spectrum obtained using a ^10^B target with 0.5 % F_2_ in **(a)** ^14^N_2_ matrix, **(b)** ^15^N_2_ matrix, and **(c)** 50 % ^14^N_2_ + 50 % ^15^N_2_ matrix. Natural boron target with 0.5 % F_2_ in **(d)** ^14^N_2_ matrix, **(e)** ^15^N_2_ matrix, and **(f)** 50 % ^14^N_2_ + 50 % ^15^N_2_ matrix.

The absorptions associated with the novel fluoroborylene : N_2_ product molecules ***A***–***C*** were unambiguously assigned based on their growth/decay characteristics in different experiments and on their characteristic ^10/11^B and ^14/15^N isotope pattern. The novel ring molecule ***A*** shows strong bands in the B‐F stretching region at 1531.0 and 1558.6 cm^−1^, which are tracked by weaker ring vibrations at 1090.7 and 1101.5 cm^−1^. These bands were already observed on deposition, they increased by 50 % on annealing to 15 K, and continue to grow on irradiation at *λ*=278±10 nm, where they reached three‐fold on further annealing to 15 K. For a natural boron (^10^B:^11^B=19.9:80.1) sample, the vibrational modes of a diboron species with two equivalent boron atoms split into three absorptions with approximately 1:8:16 (^10^B^10^B, ^10^B^11^B, ^11^B^11^B) relative intensities.^[11]^ In addition to the two B‐F stretching bands for the two most abundant isotopologues of ***A*** (Tables 1 and S3) the corresponding band associated with the ^10^B^10^B species is observed in ^10^B + F_2_/N_2_ mixture experiments at 1590.2 cm^−1^, giving a ^10^B/^11^B isotopic ratio of 1.0381. The ^11^B^11^B ring vibration observed at 1090.7 cm^−1^ shift to 1123.8 cm^−1^ (^10^B^10^B) in these experiments, indicating a ^10^B/^11^B isotopic ratio of 1.0303, a typical boron atom involved isotopic ratio. As shown in Figure 2 the boron isotopic distribution supports the presence of two equivalent boron atoms in this molecule. In experiments using F_2_/^15^N_2_ mixtures (Figure 2) the ^15^N counterpart bands were observed at 1569.6 and 1108.5 cm^−1^ (^10^B^10^B), 1542.0 cm^−1^ (^10^B^11^B) and 1521.4 and 1077.0 cm^−1^ (^11^B^11^B), respectively (Figures S1). The relative intensities of the bands due to the ^14^N and ^15^N isotopomers are almost the same in experiments using 50 % ^14^N_2_ and 50 % ^15^N_2_ mixtures (Figure 2 f). Obviously, two B atoms bind only one ^14^N_2_ or ^15^N_2_ in this new ring molecule. We also note a good agreement between the observed and calculated frequencies at the B3LYP and CCSD(T) levels of theory listed in Tables 1 and S3.

**Table 1:** Observed and calculated (CCSD(T)/def2‐TZVP) vibrational frequencies (cm^−1^) and isotopic frequency ratios (ν(^10^B)/ν(^11^B)) of the FB(*μ*‐N)_2_BF, FBNNBF and FB(*η* ^2^‐N_2_) molecules.^[a]^

	^11^B^11^B/^11^B		^10^B^11^B		^10^B^10^B/^10^B		^10^B/^11^B ratio	
	calcd^[a]^	Obs.	calcd^[a]^	Obs.	calcd^[a]^	Obs.	calcd	Obs.
cyclic FB(*μ*‐N)_2_BF (^1^A_1_)								
	*1612.7 (68)* *1538.5 (802)* 1114.6 (113)	*1612.8* *1531.0* 1090.7	*1642.2 (88)* *1546.2 (733)* 1125.4 (115)	*1635.8* *1558.6* 1101.5	*1662.9 (70)* *1585.1 (850)* 1140.5 (120)	*1660.7* *1590.2* 1123.8	1.0311 1.0302 1.0232	1.0297 1.0382 1.0303
^14^N								

	*1597.1 (57)* *1523.2 (785)* 1093.5 (110)	*hidden*	*1638.2 (87)*	/	*1659.2 (64)*	*1662.8*	1.0387	/
^15^N		*1521.4*	*1541.8 (727)*	*1542.0*	*1580.1 (844)*	*1569.6*	1.0377	1.0317
		1077.0	1110.5 (113)	/	1125.8 (118)	1108.5	1.0295	1.0292

linear FBNNBF (^1^Σ_g_^+^)								
^14^N	2068.8 (927)	2078.2	2099.0 (934)	2108.8	2138.6 (1012)	2147.0	1.0337	1.0331
	*976.1 (178)*	*981.0*	*977.4 (176)*	/	*978.9 (174)*	*983.1*	1.0029	1.0021
^15^N	2049.5(933)	hidden	2079.2 (929)	2073.4	2120.2 (1017)	2125.0	1.0345	/
	*962.7(168)*	*969.4*	*963.8 (166)*	/	*964.9 (165)*	*970.4*	1.0023	1.0010

cyclic FB(*η* ^2^‐N_2_) (^1^A_1_)								
^14^N	*1704.5(356)*	*1710.5*			*1760.6(355)*	*1765.8*	1.0330	1.0323
	1231.6(45)	1229.5			1234.9(46)	1231.0	1.0027	1.0012
^15^N	*1689.6(398)*	*1700.1*			*1752.3(340)*	/	1.0371	
	1194.8(35)	1192.5			1198.6(33)	/	1.0032

[a] Scaled frequencies using a uniform scaling factor of 0.969.^[27]^ Intensities (km mol^−1^) in parentheses. Band positions assigned to B‐F stretching modes are given in italics.

**Figure 3:**
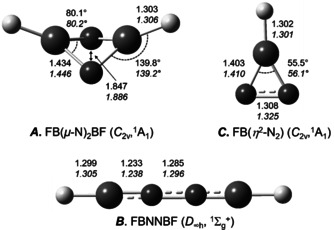
Optimized structures of ***A*** FB(*μ*‐N)_2_BF, ***B*** FBNNBF and ***C*** FB(NN) obtained at the B3LYP/6‐311++G(3df,3pd) and CCSD(T)/def2‐TZVP (*italic*) levels of theory. Bond distances are given in Å and angles in degree.

The linear isomer ***B*** (*D*_∞h_ symmetry, Figure 3) has a singlet ground state and exhibits only two infrared active vibrational modes in the mid‐IR region (Table S4, for more computational results on ***B*** see Part 1 of the Supporting Information). The antisymmetric B‐N stretching modes give rise to two bands at 2108.8 (^11^B^10^B) and 2078.2 (^11^B^11^B) cm^−1^ in Figure 1 using a natural boron target, while the antisymmetric F‐B vibration is observed only for the most abundant ^11^B^11^B isotopologue at 981.0 cm^−1^ on deposition. Experiments performed with ^10^B and N_2_ or ^15^N_2_, and ^11^B with N_2_ or ^15^N_2_ are shown in Figures 2 and S1–S3, and the observed product absorptions are compared to calculated values in Table 1 (for more details see the Supporting Information). These bands disappeared upon 273 nm irradiation, while the bands of ***A*** increased simultaneously, suggesting that isomerization is occurring. Note, linear ***B*** is isoelectronic to diisocyanate, OCNNCO.^[12]^ Like ***B*** also OCNNCO is photosensitive and decomposes rapidly under UV light to produce N_2_ + 2 CO.

Cyclic ***C*** (Figure 3) is assigned to a band at 1710.5 cm^−1^ in Figure 1, and another very small band at 1229.5 cm^−1^, which corresponds to the F‐B and N‐N stretching modes, respectively (Tables 1 and S5). Further experiments were performed using ^10^B and N_2_ or ^15^N_2_, and ^11^B with N_2_ or ^15^N_2_, and the absorptions of the corresponding isotopologues were observed at 1765.8 and 1331.0 cm^−1^ (^10^B), and at 1700.1 and 1192.5 cm^−1^ (^15^N, Figures S1,S3). These bands were observed on co‐deposition, but on annealing to 15 K they disappeared entirely, while the bands due to ***A*** increased by 30 %.

According to B3LYP/6‐311++G (3df, 3pd) calculation linear ***B*** is separated from cyclic ***A*** by a barrier of 25.0 kcal mol^−1^ and higher in energy by 22.3 kcal mol^−1^ (Figures S5, S11). At this level the predicted N‐N distance in ***A*** is 1.847 Å (CCSD(T)/def2‐TZVP: 1.886 Å), which is significantly longer than for example, the N−N single bond of diphenylhydrazine [*d*(N−N): 1.394 Å]^[5]^ and indicates a complete splitting of the N≡N triple bond by the two FB units. The computed B‐N distance (1.434 Å, Figure 3) is in the range of a conventional B=N double bond, like in aminoboranes.^[13]^ The ring inversion barrier of the puckered ring of ***A*** was found to be 15.8 kcal mol^−1^ at the B3LYP/6‐311++G(3df, 3pd) level of theory (Figure S13).

**Figure 4:**
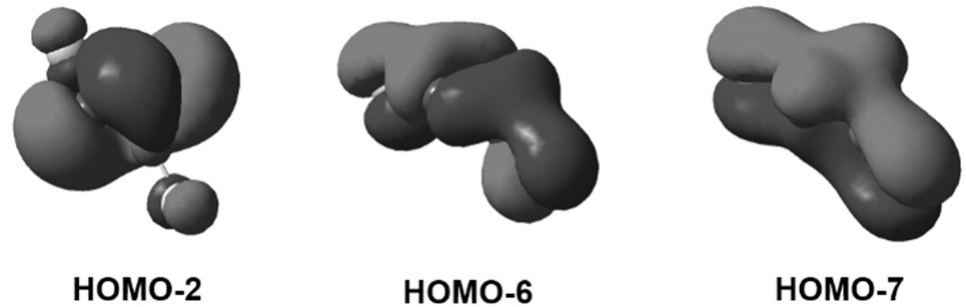
Selected frontier molecular orbitals of FB(*μ*‐N)_2_BF.

The proposed aromatic π‐electron delocalization in the cyclic compounds ***A*** and ***C*** is supported by a molecular orbital (MO) analysis and computed nucleus‐independent chemical shift (NICS) values. Figures 4 and S14 shows typical π‐bonding orbitals of the cyclic π‐conjugated system ***A***, which consists of the 4c‐2e central bonding orbital (HOMO−2) and two further π‐bonding orbitals (HOMO−6 and −7), to which the exo‐cyclic F atoms clearly contribute. Related puckered 4‐membered ring aromates have previously thoroughly been analyzed.[^14^, ^15^]

The NICS value is among the most popular aromaticity indices.^[16]^ While the NICS index was originally obtained for planar aromatic systems, it has recently been suggested to calculate an average NICS(1)_av_ index, NICS(1)_av_=[NICS(−1) + NICS(1)]/2, as a probe of aromaticity in nonplanar molecular systems.^[17]^ The large negative NICS(1)_av_ index of −21 obtained at the center of gravity of ***A*** (Figure S15,b) indicates its significant aromatic character, which can be compared to the NICS(1) value obtained for planar ***C*** of −12 (Figure S15 e).

The selective formation of the products ***A**–**C*** is surprising at first glance, but due to a high dilution of the initially formed reactive intermediates, as well as the subsequent isolation of the products in a solid N_2_ matrix at cryogenic temperatures, possible secondary reactions are efficiently suppressed. The predominant reaction of laser ablated boron atoms with N_2_ molecules^[6]^ depends on whether the boron atoms are in their ^2^P(2s^2^2p^1^) or first excited ^4^P(2s^1^2p^2^) state, located 82.5 kcal mol^−1^ higher in energy.^[18]^ Common trivalent boron compounds can usually be traced back to the first excited ^4^P state, and sub‐valent boron compounds such as Lewis‐base stabilized borylenes only recently became a rapidly emerging class of highly reactive intermediates.[^3^, ^4^] However, due to the very low Lewis basicity of N_2_ it seems that ground‐state boron atoms are reluctant to react with N_2_ molecules.[^6^, ^19a^] The dissociation energy of the weakly bound B‐NN (^2^Π) adduct with respect to B(^2^P) + N_2_ (X^1^Σ_g_^+^) is reported to be only 1.2 kcal mol^−1^.^[20]^ It is interesting that only the high‐energy isomer NBN (^2^Π) is observed in the present study (Figures 1, 2). The weakly bound B‐NN (^2^Π) adduct is almost isoenergetic with the cyclic isomer B(*η* ^2^‐N_2_) (^2^A_1_), while the linear trivalent boron isomers BNN (^4^Σ_g_^−^) and NBN (^2^Π) are substantially higher in energy by 7.7 and 22 kcal mol^−1^, respectively.^[20]^ Given that the adduct B‐NN (^2^Π) is separated from the other three isomers by significant computed energy barriers,^[19b]^ the observation of only the highest‐energy isomer NBN (^2^Π) could indicate its higher kinetic stability compared to the other isomers under the experimental conditions. A plausible route to the formation of NBN is the reaction of N_2_ molecules upon the deposition with excited ^4^P boron atoms, which are produced during laser‐ablation.

Prominent bands due to NNBN (^1^Σ_g_^+^) and BF (^1^Σ_g_^+^) in the spectra obtained after deposition (Figure 1) indicates the presence of free N and F atoms in the deposit. These atoms are commonly generated in the hot plasma plume or by photo‐decomposition of N_2_ and F_2_ molecules, respectively, as a result of the plasma broadband radiation that is produced during laser ablation. These atoms react very exothermically with boron atoms to yield diatomic NB and FB molecules, respectively, but only NB react further with N_2_ molecules during deposition to yield NNBN.^[6]^ In addition, the lack of mixed ^14^N/^15^N isotopologues of NBN in experiments using 1:1 mixtures of ^14^N_2_ and ^15^N_2_ rule out the formation of NBN from diatomic BN and N atoms, which corroborates our assumption of NBN formation by insertion of excited B atoms into N_2_ molecules.

Our search for FBNN (Figure S19) in the experimental spectra failed, and, in contrast to NBNN and PhB‐NN^[4e]^ free diatomic BF was observed. In agreement with previous results^[8]^ our calculations revealed that the linear adduct FBNN is endothermic by 23.4 kcal mol^−1^ compared to N_2_ + singlet FB (Figure S19). This observation is consistent with the larger singlet‐triplet gap of FB (+80 kcal mol^−1^)^[20a]^ compared to that of diatomic NB (−0.5 kcal mol^−1^)^[20b]^ and PhB (+31 kcal mol^−1^).^[20c]^

For the formation of the novel FB : N_2_ adducts ***A***–***C***, we have explored both the fluorination of initially formed BN_2_ intermediates and the reaction of fluoroborylene FB with N_2_ molecules (Figures S4, S5). Although the reaction of the BN_2_ species with F atoms is strongly exothermic, preliminary B3LYP/6‐311++G(3df,3pd) calculations suggest significant reaction barriers, for example, 14 kcal mol^−1^ for the reaction of NBN (^2^Π) with F atoms (Figure S4). These calculations also revealed that cyclic ***C*** is higher in energy by 8.1 kcal mol^−1^, but kinetically stable to its decomposition into N_2_ + singlet FB due to a barrier of 42 kcal mol^−1^ (Figure S5).

**Figure 5:**
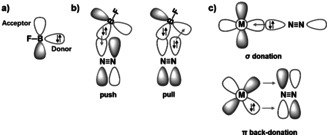
**a)** Scheme of the FB acceptor and donor orbitals. Note that FB has two mutually perpendicular π‐acceptor orbitals, only one of which is shown. **b)** Bonding interaction in the transition state of the FB + N_2_ reaction, **c)** *end‐on* complex of N_2_ to a transition metal M, see Figure S12.

Activation of N_2_ molecules and weakening of its π bonds can mainly be attributed to interactions that donate electron density to its π* antibonding orbitals, and remove electron density from the π bonding orbitals of N_2_.^[21]^ Unlike transition metal complexes, which provide a σ acceptor and a π donor orbital to form N_2_ complexes (Figure 5 c), singlet FB can be viewed as an electrophilic σ donor and π acceptor (Figure 5 a). Its side‐on attack on N_2_ (Figures 5 c and S12) enables electron donation from its sigma donor orbital into the π* MO of N_2_ and removal of π‐bonding electrons into the π* MO of BF. These bonding interactions are supported by an energy decomposition analysis (EDA).^[22]^ This shows that the two major orbital interactions Δ*E*_orb_(1) and Δ*E*_orb_(2), which contribute with −119.3 and −35.0 kcal mol^−1^ to the total orbital term Δ*E*_orb_ in the transition state of the FB + N_2_ reaction, can be attributed to σ(FB) donation and π(N_2_) back‐donation, respectively (Figure S20). Since the reaction between boron and fluorine atoms is strongly exothermic (182 kcal mol^−1^),^[20a]^ this reaction energy can provide the activation energy for the singlet FB + N_2_ during the deposition of the matrix. It can, however, not be excluded that ***C*** can also be produced in an exothermic reaction of N_2_ molecules with triplet excited FB (^3^Π), which is 80 kcal mol^−1^ higher in energy than the singlet ground‐state,[^20a^, ^20c^] and likely formed by UV radiation (λ<357 nm) emitted from the plasma plume.

We recall that ***C*** is observed only in freshly deposited samples, and it disappeared already upon annealing to 15 K. Cyclic ***C*** is isolectronic to the known diazirinone, OC(*η* ^2^‐N_2_),^[23a]^ and although ***C*** should be more stable because of a higher barrier and a lower dissociation energy,^[23b]^ the N=N bond in ***C*** is strongly activated and it reacts readily with a second singlet FB molecule to yield ***A*** through a very low energy barrier of 0.5 kcal mol^−1^(Figure S5).

Since B_2_N_2_ isomers were barely observed, they can hardly be considered as starting compounds for the bisfluoroborylene: N_2_ compounds ***A*** and ***B***. However, ***A*** and ***B*** are most likely formed in an exothermic and low‐barrier reaction from BF dimer molecules and N_2_ (Figure S5). The two lowest energy BF dimer isomers have been considered, the linear triplet FB=BF structure (^3^Σ_g_^−^) and a singlet *trans*‐bent isomer of *C*_2h_ symmetry in a ^1^A_g_ ground state (Table S8). In contrast to the isoelectronic CO dimer, OCCO (^3^Σ_g_^−^),^[24]^ both of these BF dimers are more favourable than the diatomic fragments in their ground singlet state. The linear triplet FBBF, which formally arises from a double σ→π* excitation of the two singlet BF fragments,^[8]^ is more stable than the *trans*‐bent isomer by 8 kcal mol^−1^ (Table S8). On the other hand, the *trans*‐bent isomer could be formed through mutual σ→π donor‐acceptor interactions of two ground‐state singlet BF molecules via a loose and low‐energy (<1.0 kcal mol^−1^) *C*_2h_ symmetric transition state.^[8]^ Since the computed antisymmetric B‐F stretching frequencies of these two isomers are similar (Table S8), our assignment of the experimentally observed B‐F stretching frequencies to the *trans*‐bent isomer (Table S8) is therefore very tentative and only supported by its predicted low‐barrier formation from two singlet BF molecules. Nevertheless, the positive dissociation energy and the low barrier of formation of *trans*‐bent FBBF from two BF molecules combined with a low barrier for the subsequent reaction with N_2_ molecules of 10 kcal mol^−1^ at the B3LYP/6‐311++G(3df, 3pd) level provide a surprisingly selective, low‐barrier route to the title compound ***A*** on the singlet potential energy surface (Figure S5).

It was shown that the parent diborene, HB=BH, is efficiently stabilized by Lewis base ligands L to yield planar adducts L(H)B=B(H)L, with L=CO^[25]^ or bulky carbene ligands.^[26]^ Preliminary calculations at the B3LYP/6‐311++g(3df,3pd) level indeed predict that also the addition of the weak N_2_ donor molecule to FBBF would be strongly exothermic and yield the corresponding adducts FB=B(N_2_)F (*C*_s_, Δ*E*=−25.6 kcal mol^−1^) and F(N_2_)B=B(N_2_)F (*C*_2h_, Δ*E*=−34.7 kcal mol^−1^, Table S9 and S10). However, we found no spectroscopic evidence for the predicted formation of these dinitrogen adducts of FBBF in the cryogenic N_2_‐matrix and we note that the adduct FB=B(N_2_)F is still significantly higher in energy than the experimentally observed isomers ***A*** and ***B*** by 44 and 22 kcal mol^−1^, respectively. The cyclic compound ***A*** is both energetically (Figures S4, S5) and kinetically very stable, so that it can be expected that, like the analogous diazirinone, OC(*η* ^2^‐N_2_),^[23a]^ it could be viable also at ambient conditions.

## Conclusion

In conclusion, the two novel cyclic fluorodiazaboririne, FB(*η* ^2^‐N_2_) (***C***), and 1,3‐Diaza‐2,4‐diborete, (FB)_2_N_2_ (***A***), as well as the linear compounds FB=BF and FBNNBF (***B***) were produced from laser‐ablated boron atoms and fluorine embedded in an excess of N_2_. The aromatic nature of the electron‐deficient rings of ***A*** and ***C***, reinforced by fluorine specific interactions based on electronic contribution from the π lone pairs of the exo‐cyclic fluorine atoms, and confirmed by an MO analysis and computation of their NICS index, contributes to their high thermodynamic stability. Their surprisingly selective formation can be traced back to the high reactivity of fluoroborylene intermediates. This work may contribute to exciting applications in dinitrogen fixation and activation.

## Conflict of interest

The authors declare no conflict of interest.

## Supporting information

As a service to our authors and readers, this journal provides supporting information supplied by the authors. Such materials are peer reviewed and may be re‐organized for online delivery, but are not copy‐edited or typeset. Technical support issues arising from supporting information (other than missing files) should be addressed to the authors.

Supplementary
