# Supplementary material for: Cleavage of the N≡N Triple Bond and Unpredicted Formation of the Cyclic 1,3‐Diaza‐2,4‐Diborete (FB)2N2 from N2 and Fluoroborylene BF
Source: Angew Chem Int Ed Engl. 2021 Jun 26;60(31):17205–10. doi: 10.1002/anie.202106984 (PMC8361949; doi:10.1002/anie.202106984)
Supplement: Supplementary file 1 — Supplementary [file ANIE-60-17205-s001.pdf]

Angewandte Chemie
Eine Zeitschrift der Gesellschaft Deutscher Chemiker

Supporting Information

Cleavage of the N≡N Triple Bond and Unpredicted Formation of the Cyclic 1,3-Diaza-2,4-Diborete $(FB)_2N_2$ from N_2 and Fluoroborylene BF

Bing Xu, Helmut Beckers, Haoyu Ye, Yan Lu, Juanjuan Cheng, Xuefeng Wang, and Sebastian Riedel**

anie_202106984_sm_miscellaneous_information.pdf

Table of Contents

Part 1: Exp. Details, Comp. Methods and Details about the Spectra Page 1
Experimental Details Page 1
Computational Methods Page 1
Spectral Assignment of FBNNBF Page 1
Computational results for FBNNBF Page 2
References to Part 1 Page 3

Part 2: Supporting Figures Page 4
Figure S1. Isotopic ^{10}B target with 0.5% F_2 in N_2 matrix. Page 4
Figure S2. Isotopic ^{10}B target with 0.5% F_2 in $^{15}N_2$ matrix. Page 4
Figure S3. Natural boron target with 0.5% F_2 in $^{15}N_2$ matrix. Page 5
Figure S4. Computed reaction coordinates for reactions of NBN + F to yield FB(μ-N)$_2$BF via cyclic FB(μ-N)$_2$. Page 6
Figure S5. Computed reaction coordinates for reactions of FB + N_2 to yield FB(μ-N)$_2$BF. Page 7
Figure S6. Transition state structure (TS1) (^{1}A, C_1) and its frequencies. Page 7
Figure S7. Transition state structure (TS2) (^{3}A, C_1) and its frequencies. Page 7
Figure S8. Transition state structure (TS3) (^{3}A, C_1) and its frequencies. Page 8
Figure S9. Transition state structure (TS4) (^{3}A, C_1) and its frequencies. Page 9
Figure S10. Transition state structure (TS5) (^{1}A, C_1) and its frequencies. Page 9
Figure S11. Intrinsic reaction coordinate (IRC) via the transition state TS (^{1}A, C_1, TS5 in Figure S4) for the mutual isomerization. Page 10
Figure S12. Transition state structure (TS6) (1A, C1) and its frequencies obtained at the B3LYP/6-311++G(3df, 3pd) level of theory. Page 10
Figure S13. Transition state for the ring inversion of FB(μ-N)$_2$BF ($^1A_1$, D_{2h}) and its frequencies. Page 11
Figure S14. Selected frontier molecular orbitals of FB(μ-N)$_2$BF calculated at the B3LYP/6-311++G(3df, 3pd) level. Page 11
Figure S15. NICS values of the nonplanar cyclic-BNBN ring calculated at the B3LYP/aug-cc-pvtz level of theory. Page 12
Figure S16. Selected frontier molecular orbitals of FBNNBF. Page 12
Figure S17. MO scheme and frontier molecular orbitals (isovalue=0.04 a.u.) of singlet state FB(μ-N)$_2$BF and FBNNBF. Page 13
Figure S18. Selected frontier molecular orbitals of FB(η^2-N_2) Page 14
Figure S19. Computed oligomers of BF and N_2. Page 14
Figure S20. Plot of the deformation densities Δρ of the BF→N_2 donation and N_2→BF back-donation in TS6 Page 15

Part 3: Supporting Tables Page 16
Table S1. Experimental frequencies of binary boron nitrides. Page 16
Table S2. Experimental frequencies of binary boron fluorides. Page 16
Table S3. Observed and calculated fundamental frequencies of FB(μ-N)$_2$BF. Page 17
Table S4. Observed and calculated fundamental frequencies of FBNNBF. Page 18
Table S5. Observed and calculated fundamental frequencies of FB(NN) Page 18
Table S6. Computed structures of FBNNBF and FB(μ-N)$_2$BF Page 19
Table S7. Calculated fundamental frequencies of FB(η^2-N_2) and FBNN. Page 19
Table S8. Observed and calculated fundamental frequencies of linear FBBF ($D_{\infty h}$, ^{3}B) and bent FBBF (C_{2h}, ^{1}A). Page 19
Table S9. Calculated relative energies for the lowest triplet and singlet states of $(BF)_2(N_2)_n$ Page 20
Table S10. Computed fundamental frequencies of FB=B(N_2)F (C_s, ^{1}A′) and F(N_2)B=B(N_2)F Page 20

Total Number of Pages-20

Part 1: Experimental Details, Computational Methods and Details about the Spectral Assignment of FBNNBF

Experimental Details

BF was produced by a laser ablated boron target (Alfa Aesar) reacting with F_2 gas in excess nitrogen.[1] Nitrogen serves as reactant as well as matrix host. Although BF, BF_2 and BF_3 were produced in the reaction of ablated boron atoms with F_2 simultaneously, we keep the F_2 dilution in N_2 gas less than 1% and the flow speed of the F_2/N_2 gas mixtures no more than 2 mmol/h in order to optimize the formation of BF. The products of the FB reaction with N_2 were distributed uniformly onto the CsI matrix support. Infrared spectra (0.5 cm^{-1} resolution) were recorded using a Bruker Vertex 70 spectrometer, and the deposits were later annealed or irradiated by using LEDs and a high pressure mercury lamp to allow reagent diffusion and further reaction.

Computational Methods

Theoretical calculations were performed to predict the structures and vibrational frequencies of the reaction products by using the Gaussian 09 program.[2] DFT calculations were performed using the B3LYP hybrid functional[3, 4] in conjunction with the 6-311++G(3df, 3pd) basis set for boron, fluorine and nitrogen.[5] CCSD(T)/def2-TZVP calculations were also performed.[6,7] In addition, ab initio calculations based on high-level multi-configurational wavefunction methods were used to obtain accurate electronic structure information of the FB(μ-N)$_2$BF and FBNNBF molecules using the ORCA 4.0.1 program.[8, 9] CASSCF calculations [10] including fourteen active electrons in fourteen active orbitals [CAS(14e, 14o)] were performed with def2-QZVP basis set. The EDA-NOCV [11] calculations were carried out with the ADF 2017 program package [12,13] to investigate the nature of nitrogen-boron interactions in the $F_2B_2N_2$ molecules.

Spectral Assignment of FBNNBF

The new bands observed at 2078.2 and 2108.8 cm^{-1} with 2:1 relative intensity using boron in natural abundance showed a similar behavior in annealing and photolysis experiments (see main text Figure 1). In $^{10}B + F_2/N_2$ experiments a weak band at 2147.0 cm^{-1} revealed a similar behavior (Figure 2). Obviously the 2108.9 cm^{-1} band between 2147.0 and 2078.2 cm^{-1} is due the $^{10}B/^{11}B$ isotopologue and the isotopic pattern as well as its ratio of 1.0331 suggests that two boron atoms are involved in this mode. In $F_2/^{15}N_2$ experiments the counterpart appeared at 2125.0 cm^{-1} (^{10}B) and the band due to the ^{11}B isotopologue was hidden by the stronger $B(NN)_x$ band. The band at 2073.4 cm^{-1} is assigned to the mixed $^{10}B,^{11}B$ isotopologue. These group **B** bands are appropriate for the linear FBNNBF molecule.

Computational Results for FBNNBF

The DFT and CCSD(T) calculations predict for FBNNBF ($D_{\infty h}$) a singlet ground electronic state. The ^{11}B–N antisymmetric stretching frequency, predicted at 2140.3 cm^{-1} (DFT) and 2148.8 cm^{-1} (CCSD(T), Table S4), is overestimated by 2.9% (62.1 cm^{-1}) and 3.4% (70.6 cm^{-1}), respectively. The computed N-N bond length (1.285 Å) is much longer than that of a NN triple bond (1.08 Å) and even longer than a NN double bond (1.20 Å).[14] The B-N bond length was calculated to be 1.233 and 1.238Å at the B3LYP/6-311++G(3df,3pd) and the CCSD(T)/def2-TZVP level, respectively, which is even shorter than the predicted triple B≡N distance (1.27 Å) [14] and comparable to the computed bond length in MeN=BMe obtained by MP2 and DFT calculations,[15] and in the *tert*-butyl analogue *t*-BuNB*t*-Bu (1.258Å) obtained by X-ray diffraction analysis.[16] The relative energy for FBNNBF molecule is 22.2 kcal mol^{-1} higher than that of FB(μ-N)$_2$BF. The frontier orbitals of linear FBNNBF (Figure S16) shows a degenerate π_g HOMO, which is primarily B-N π bonding and a degenerate π_u HOMO-1, which is B-N-N-B π bonding with four-center-two electrons character each. The HOMO-2 is N-N

σ and B-N σ bonding. The Lewis structure can be drawn as F-B$^{\ominus}$≡N$^{\oplus}$–N$^{\oplus}$≡B$^{\ominus}$ -F. The Lewis structure suggest a B≡N triple bond and an N–N single bond, which is in good agreement with the predicted vibrational stretching frequencies of about 2100 cm^{-1} (B≡N) and 560 cm^{-1} (N–N, Table S4). The F atom is not involved in the B–N bonding and the antisymmetric F–B stretching mode is due to strong vibrational coupling found at an unusual low frequency at 981.0 cm^{-1} (Table S4).

References to Part 1

[1] B. Xu, L. Li, Z. Pu, W. J. Yu, W. J. Li, X. F. Wang, *Inorg. Chem.* **2019**, *58*, 2363-2371.

[2] M. J. Frisch, G. W. Trucks, H. B. Schlegel, G. E. Scuseria, M. A. Robb, J. R. Cheeseman, G. Scalmani, V. Barone, B. Mennucci, G. A. Petersson, et al. Gaussian 09, revision B.01; Gaussian, Inc. Wallingford, CT, **2009**.

[3] A. D. Becke. *J. Chem. Phys.* **1993**, *98*, 5648-5652.

[4] C. Lee, W. Yang, R. G. Parr, *Phys. Rev. B: Condens. Matter Mater. Phys.* **1988**, *37*, 785-789.

[5] F. Weigend, R. Ahlrichs, *Phys. Chem. Chem. Phys.* **2005**, *7*, 3297-3305.

[6] K. Raghavachari, G. W. Trucks, J. A. Pople, *Chem. Phys. Lett.***1989**, *157*, 479-483.

[7] R. J. Bartlett, M. Musial, *Rev. Mod. Phys.* **2007**, *79*, 291-352.

[8] F. Neese, *Wiley Interdiscip. Rev.: Comput. Mol. Sci.***2018**, 8.

[9] F. Neese, The ORCA Program System. *Wiley Interdiscip. Rev.: Comput. Mol. Sci.* **2012**, *2*, 73-78.

[10] B. O. Roos, Advances in Chemical Physics; Ab Initio Methods in Quantum Chemistry - II; K. P. Lawley, Ed.; John Wiley & Sons Ltd.: New York, **1987**; Chapter 69, 399-446.

[11] L. Zhao, M. V. Hopffgarten, D. M. Andrada, G. Frenking, *Wiley Interdisciplinary Reviews: Computational Molecular Science.* **2018**, 8 (3), e1345.

[12] ADF2017, SCM, Theoretical Chemistry; Vrije Universiteit: Amsterdam, The Netherlands. Available at: http://www.scm.com.

[13] M. P. Mitoraj, A. Michalak, *Organometallics.* **2007**, *26*, 6576-6580.

[14] P. Pyykko, *J. Phys. Chem. A* **2015**, *119*, 2326−2337.

[15] K. A. Ostby, G. Gundersen, A. Haaland, H. Noth, *Dalton Trans.***2005**, 2284-2291.

[16] P. Paetzold, C. von Plotho, G. Schmid, R. Boese, B. Schrader, D. Bougeard, U. Pfeiffer, R. Gleiter, W. Schafer, *Chem. Ber.***1984**, 117, 1089-1102.

Part 2: Supporting Figures

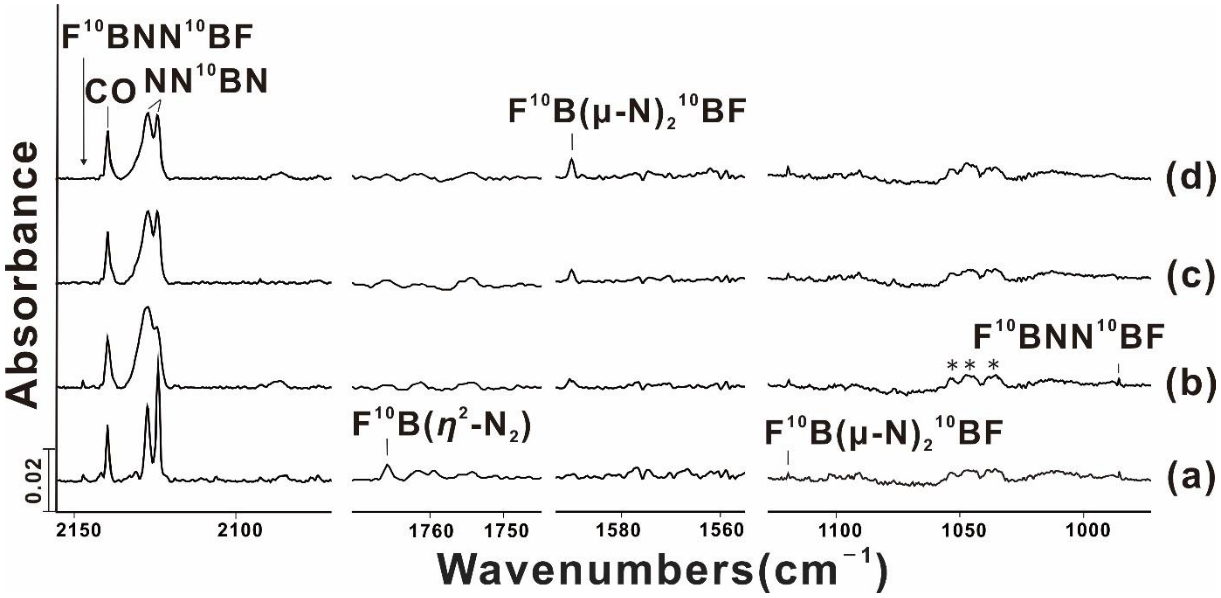

Figure S1. Excerpts from the IR spectrum obtained using an isotopic ^{10}B target with 0.5% F_2 in N_2 matrix: (a) co-deposition of B + 0.5% F_2 for 120 min, (b) after annealing to 15 K, (c) subsequent λ = 273 nm irradiation for 30 min, and (d) further annealing to 15 K. Unknown species are indicated by asterisks.

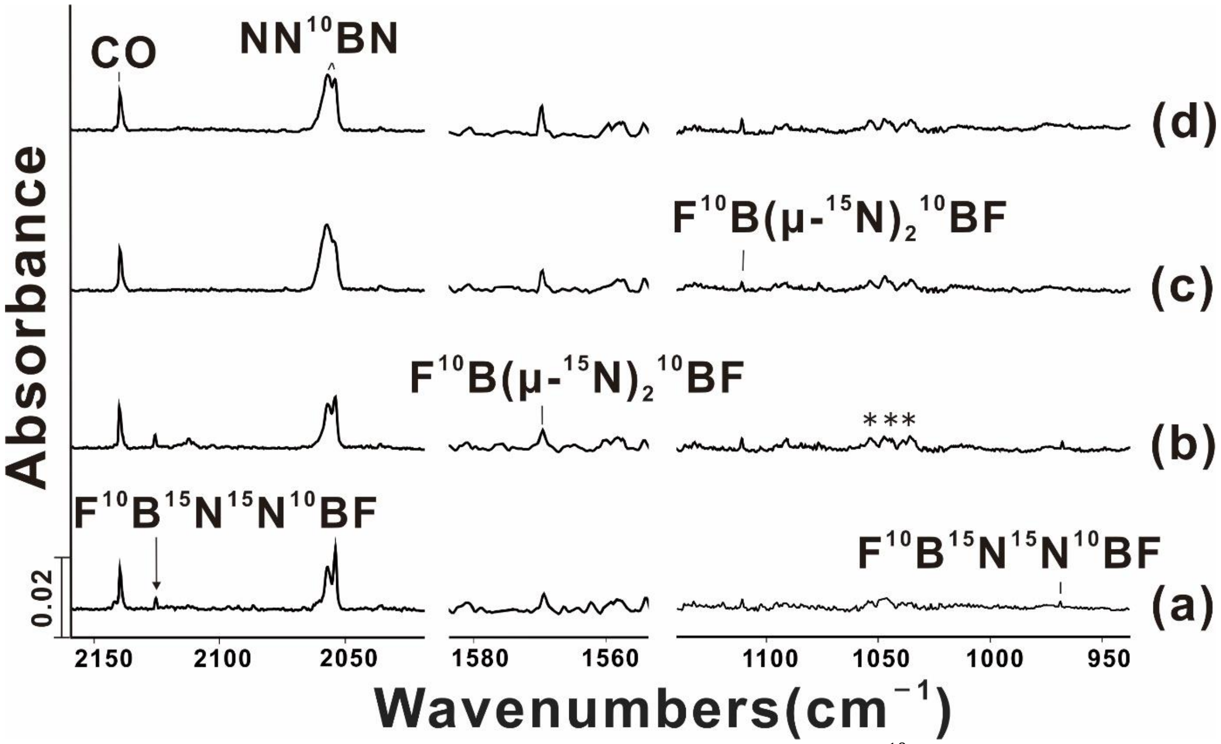

Figure S2. Excerpts from the IR spectrum obtained using an isotopic ^{10}B target with 0.5% F_2 in $^{15}N_2$ matrix: (a) co-deposition of B + 0.5% F_2 for 120 min, (b) after annealing to 15 K, (c) subsequent λ = 273 nm irradiation for 30 min, and (d) further annealing to 15 K. Unknown species are indicated by asterisks.

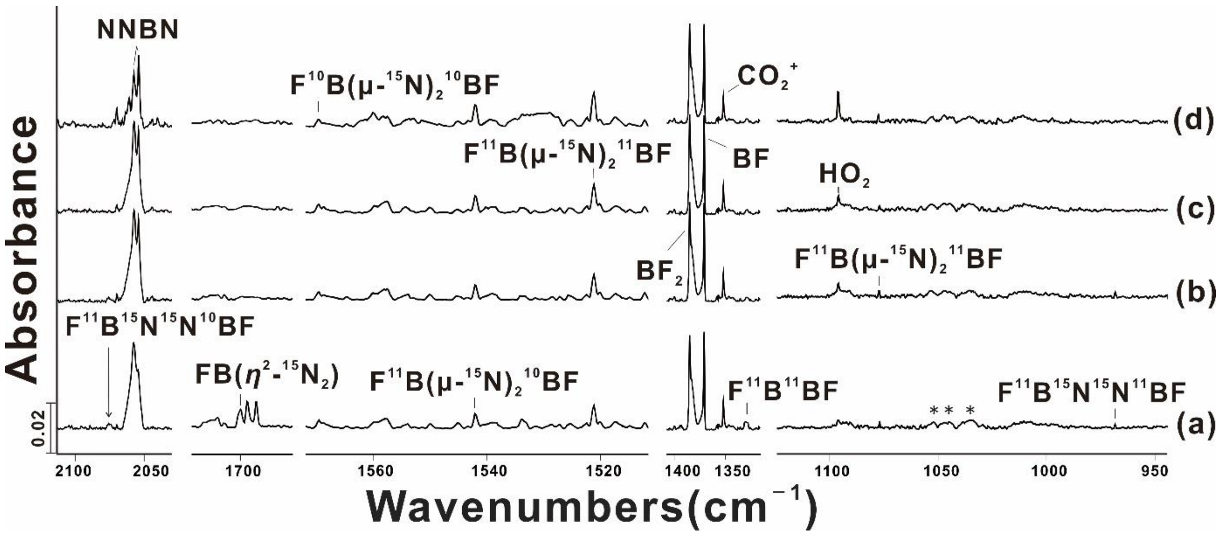

Figure S3. Excerpts from the IR spectrum obtained using a natural boron target with 0.5% F_2 in $^{15}N_2$ matrix: (a) co-deposition of B + 0.5% F_2 for 120 min, (b) after annealing to 15 K, (c) subsequent λ = 273 nm irradiation for 30 min, and (d) further annealing to 15 K. Unknown species are indicated by asterisks.

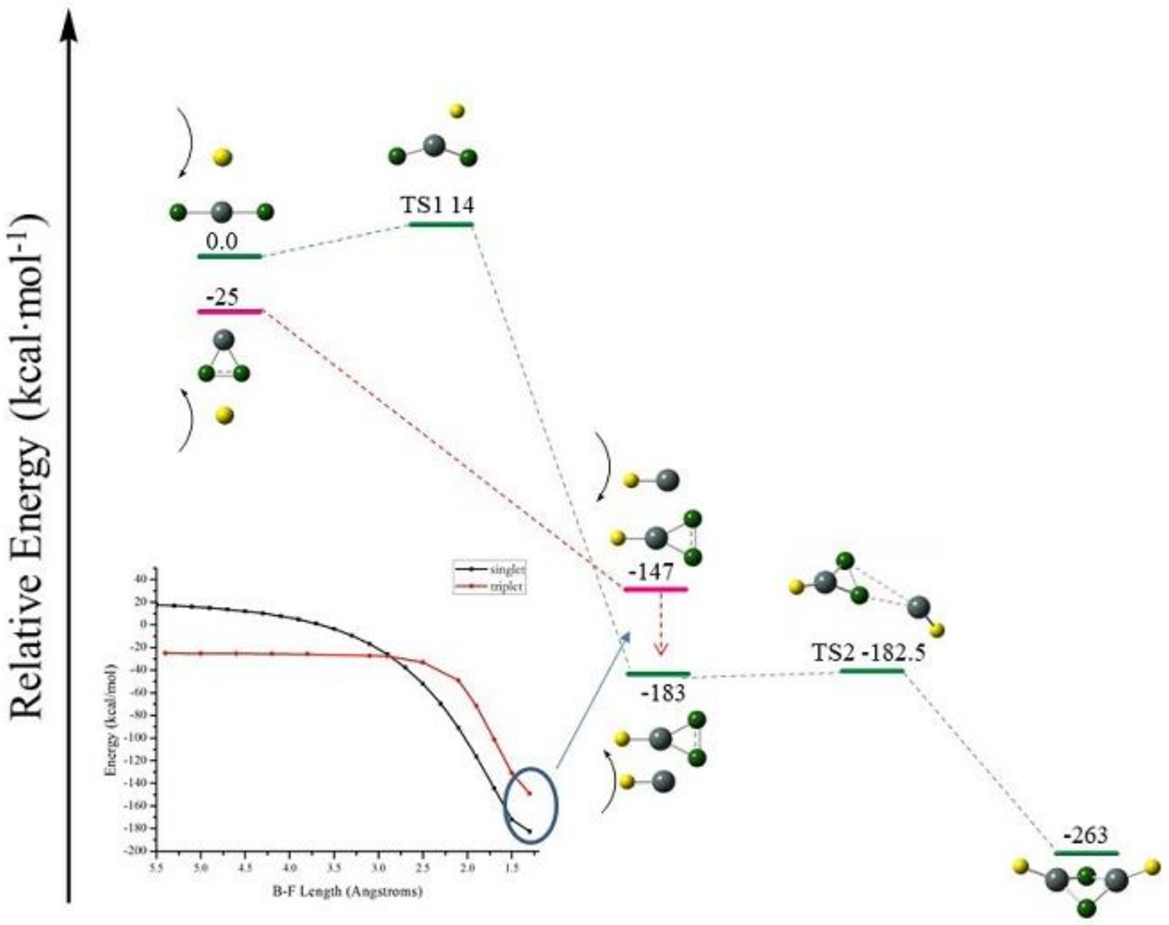

Figure S4. Computed reaction coordinates at the B3LYP/6-311++G(3df, 3pd) level for the reactions of NBN + F to yield FB(μ-N)$_2$BF via the cyclic FB(η^2-N$_2$) intermediate. Relative energies are indicated in kcal mol^{-1}. Red bars denote stationary points on the triplet surface and green bars on the singlet surface. The transition state structures TS1 and TS2 and their vibrational frequencies are shown in Figures S1 and S2, respectively. The computed intrinsic reaction coordinate (IRC) on the singlet and triplet surface for the barrier-less cleavage of the B–F bond in cyclic FB(η^2-N$_2$) to yield F + B(η^2-N$_2$) is shown at the bottom left. These coordinates indicate a crossing between the two potential surfaces.

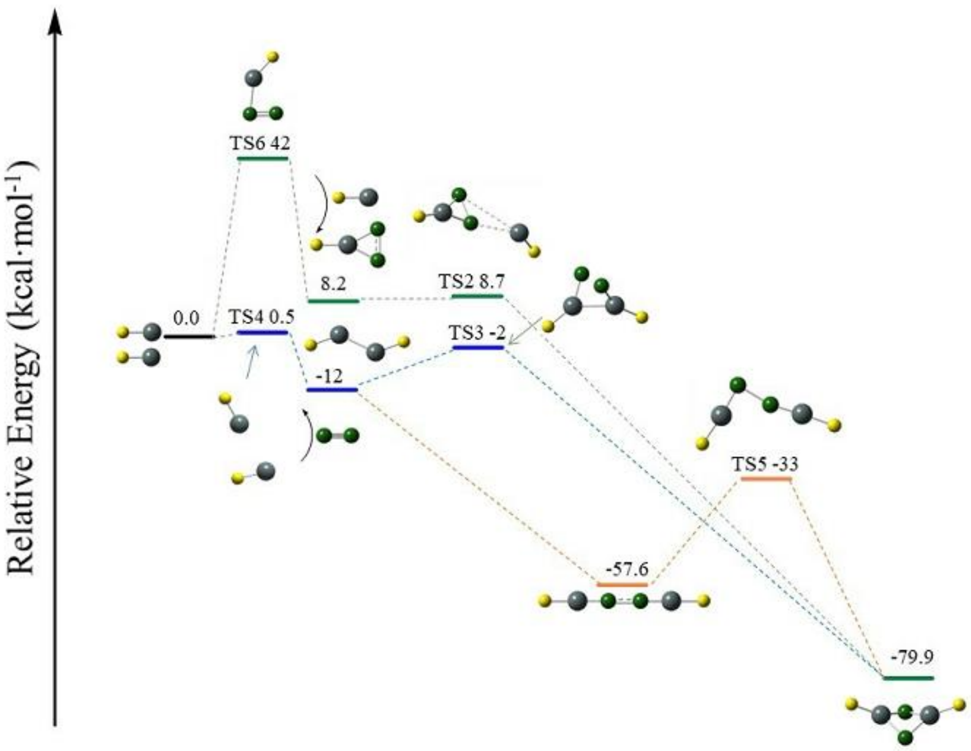

Figure S5. Computed reaction coordinates at the B3LYP/6-311++G(3df, 3pd) level for the reactions of N_2 with 2 BF to yield FBNNBF and FB(μ-N)$_2$BF and of their mutual isomerization. Relative energies are indicated in kcal mol^{-1}. Transition state structures and their vibrational frequencies for TS2-6 are shown in Figures S7 – S10 and S12, respectively. The intrinsic reaction coordinate (IRC) via the transition state TS5 is shown in Figure S11.

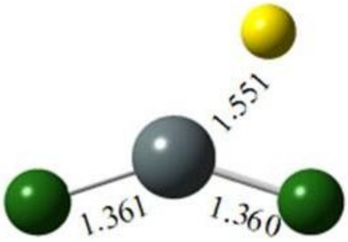

Frequency	Intensity
-445.4	20
225.6	29
384.4	20
627.5	133
1030.4	18
1578.2	24

TS1

Figure S6. Transition state structure (TS1) (^{1}A, C_1) for the reaction of linear NBN + F (Figure S4) and its frequencies (cm^{-1}) obtained at the B3LYP/6-311++G(3df, 3pd) level of theory.

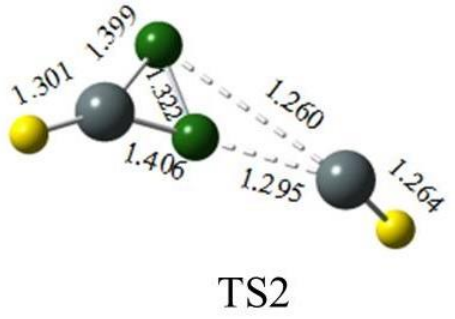

Frequency	Intensity
-150.85	1
47.5	1.5
95.3	4.3
183.1	7.1
423.0	18
490.9	85.6
788.3	3.6
1152.7	41
1238.5	172.4
213.1	213.1
1753.8	361.6

Figure S7. Transition state structure (TS2) (^{1}A, C_1) for the reaction of cyclic FB(η^2-N_2) + BF (Figures S4, S5) and its frequencies (cm^{-1}) obtained at the B3LYP/6-311++G(3df, 3pd) level of theory.

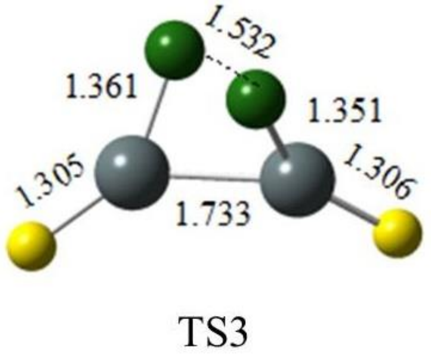

Frequency	Intensity
-506.0	29
181.5	0.5
280.2	19
373.5	0
486.3	69
519.9	57
531.4	19
768.3	14
1012.0	50
1030.9	174
1637.2	427
1696.3	159

Figure S8. Transition state structure (TS3) (^{1}A, C_1) for the reaction of *trans-bent* FBBF + N_2 (Figure S5) and its frequencies (cm^{-1}) obtained at the B3LYP/6-311++G(3df, 3pd) level of theory.

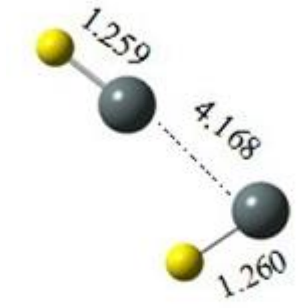

TS4

Frequency	Intensity
-23.4	2
27.9	0
32.4	1.5
52.1	3
1407.6	153
1411.4	147

Figure S9. Transition state structure (TS4) (^{3}A, C_1) for the formation of *trans-bent* FBBF from 2 BF (Figure S5) and its frequencies (cm^{-1}) obtained at the B3LYP/6-311++G(3df, 3pd) level of theory.

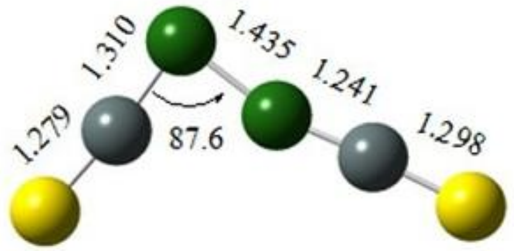

TS5

Frequency	Intensity
-325.41	155.4
94.1	2.2
185.4	0.5
369.5	18
422.2	38
428.9	43
660.7	0.5
964.2	55
1170.2	80
1884.1	405
2134.7	475.0

Figure S10. Transition state structure (TS5) (^{1}A, C_1) for the mutual isomerization of linear FBNNBF and cyclic FB(μ-N)$_2$BF (Figure S5) and its frequencies (cm^{-1}) obtained at the B3LYP/6-311++G(3df, 3pd) level of theory.

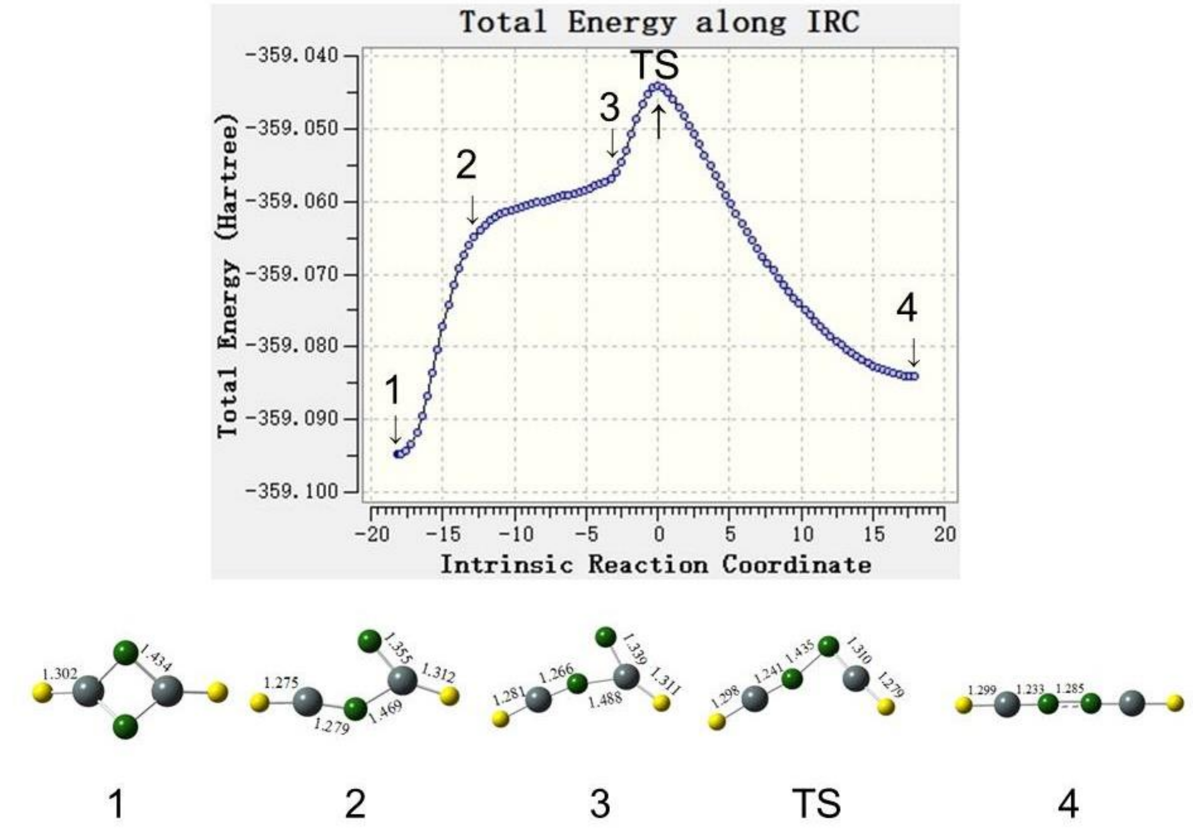

Figure S11. Intrinsic reaction coordinate (IRC) via the transition state TS5 (^1A, C_1, Figure S4) for the mutual isomerization of linear FBNNBF and cyclic FB(μ-N)$_2$BF obtained at the B3LYP/6-311++G(3df, 3pd) level of theory.

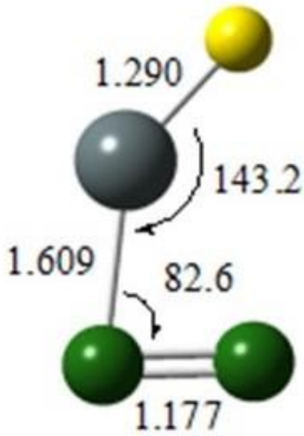

Frequency	Intensity
-766.8	88
258.5	2
377.3	25
633.3	83
1398.6	139
1813.4	312

Figure S12. Transition state structure (TS6) (^1A, C_1) for the reaction of FB + N_2 to yield cyclic FB(η^2-N_2) (Figure S5) and its frequencies (cm^{-1}) obtained at the B3LYP/6-311++G(3df, 3pd) level of theory.

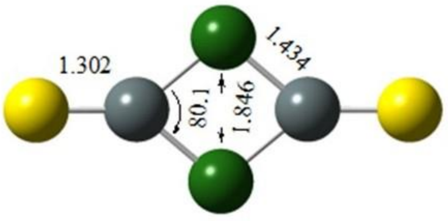

E_{rel} **:15.8**

Frequency	Intensity
-479.6	201.4
288.9	69.4
468.6	0.0
531.4	0.1
534.8	0.5
907.2	0.1
915.4	0.0
984.9	167.9
1565.8	1259.5
1653.8	0.8

Figure S13. Transition state for the ring inversion of FB(μ-N)$_2$BF ($^1A_1$, D_{2h}) and its frequencies (cm^{-1}) obtained at the B3LYP/6-311++G(3df, 3pd) level of theory. The relative energy is 15.8 kcal mol^{-1} with respect to the puckered ring.

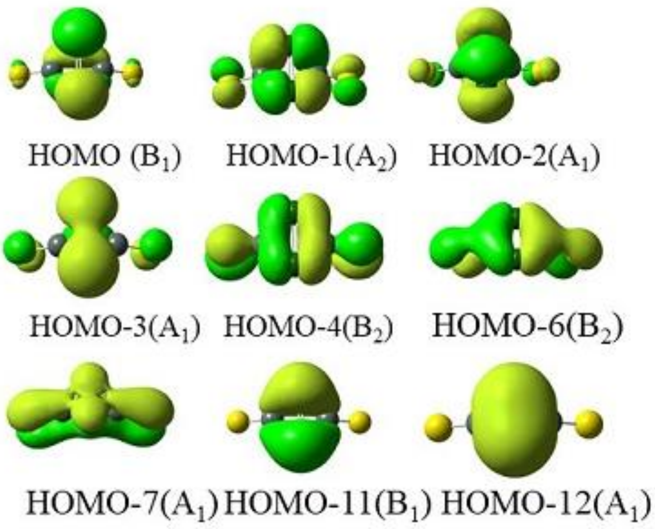

Figure S14. Selected frontier molecular orbitals of FB(μ-N)$_2$BF calculated at B3LYP/6-311++G(3df, 3pd) level.

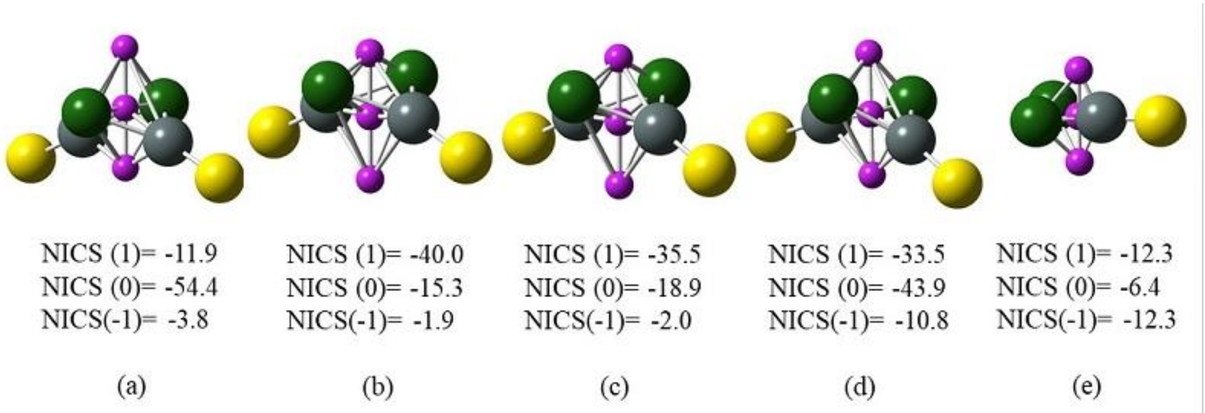

Figure S15. NICS values of the nonplanar cyclic-BNBN ring calculated at the B3LYP/aug-cc-pVTZ level of theory and obtained at the ring center (NICS(0)), 1Å above (NICS(1), and 1Å below the ring center (NICS(−1)) of the nonplanar cyclic-BNBN ring: (a) at the center of the NN linkage; (b) at the center of gravity of the molecule; (c) at center of the BB linkage; and (d) at the ring critical point (3, 1) of the B_2N_2 ring. The (e) is the NICS values of planar FB(η^2-N_2) at the ring critical point (3, 1) of the BN_2 ring.

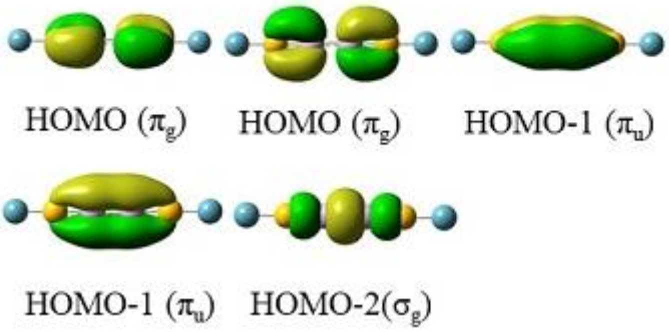

Figure S16. Selected frontier molecular orbitals of FBNNBF calculated at B3LYP/6-311++G(3df, 3pd) level.

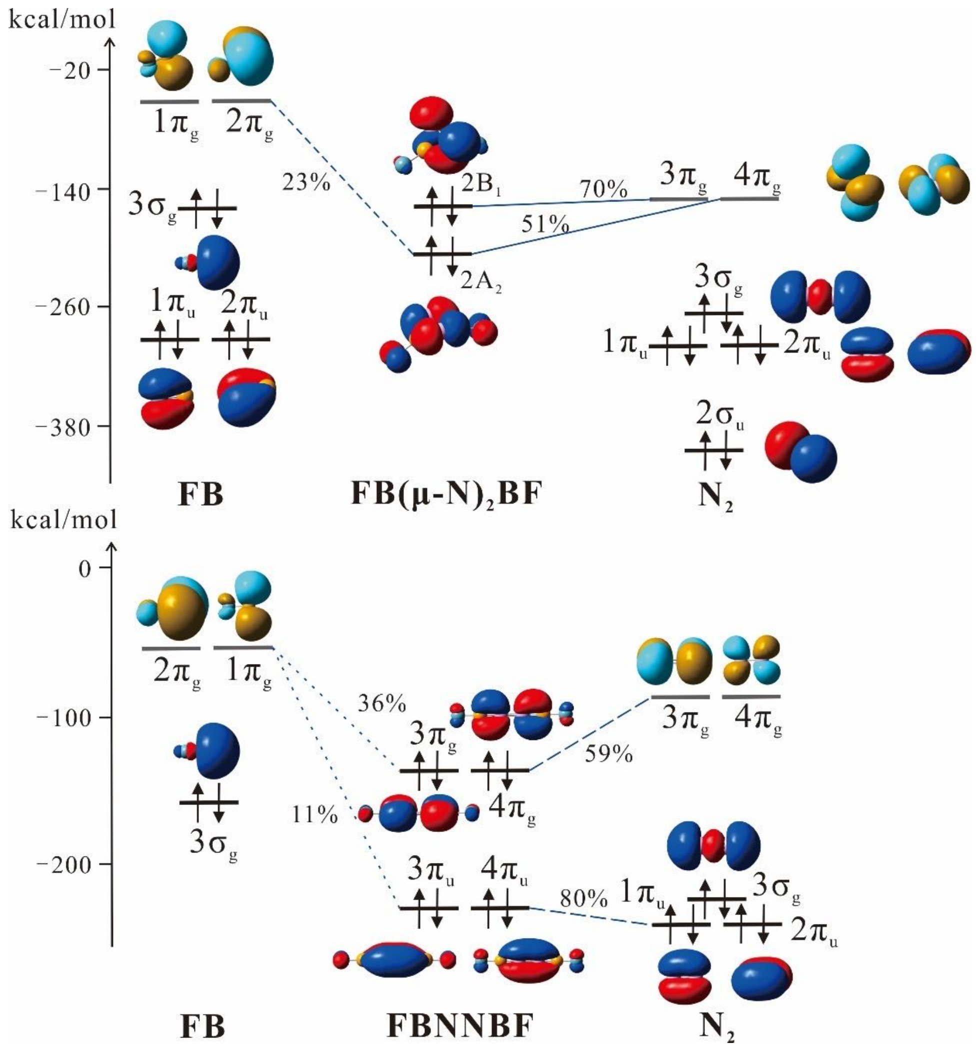

Figure S17. Frontier molecular orbitals (FMOs, isovalue = 0.04 a.u.) of singlet state cyclic FB(μ-N)$_2$BF (top), linear FBNNBF (bottom) and their constituents fluoroborylene FB and N_2. The composition of the molecular orbitals of the dinitrogen complexes are indicated in percent contributions of the fragment orbitals of BF and N_2. Note that the antibonding π MOs of the constituents fluoroborylene FB and N_2 are involved in the newly formed B–N bonding orbitals of the products.

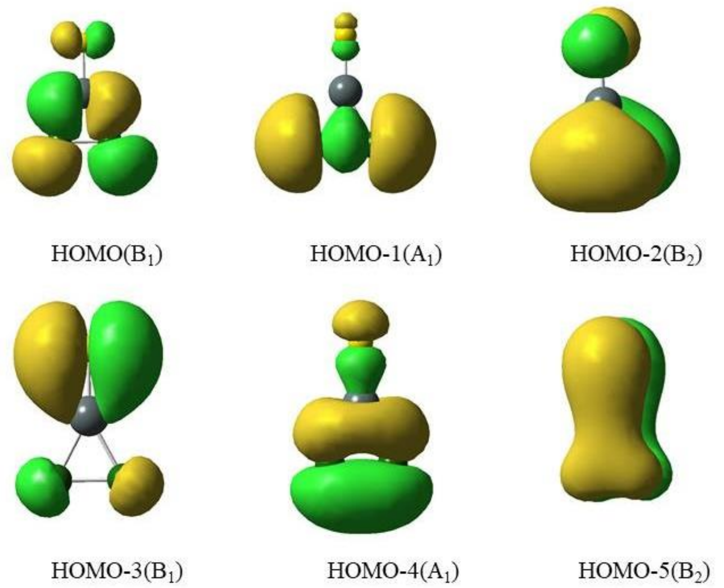

Figure S18. Selected frontier molecular orbitals of FB(η^2-N_2) calculated at B3LYP/6-311++G(3df, 3pd) level.

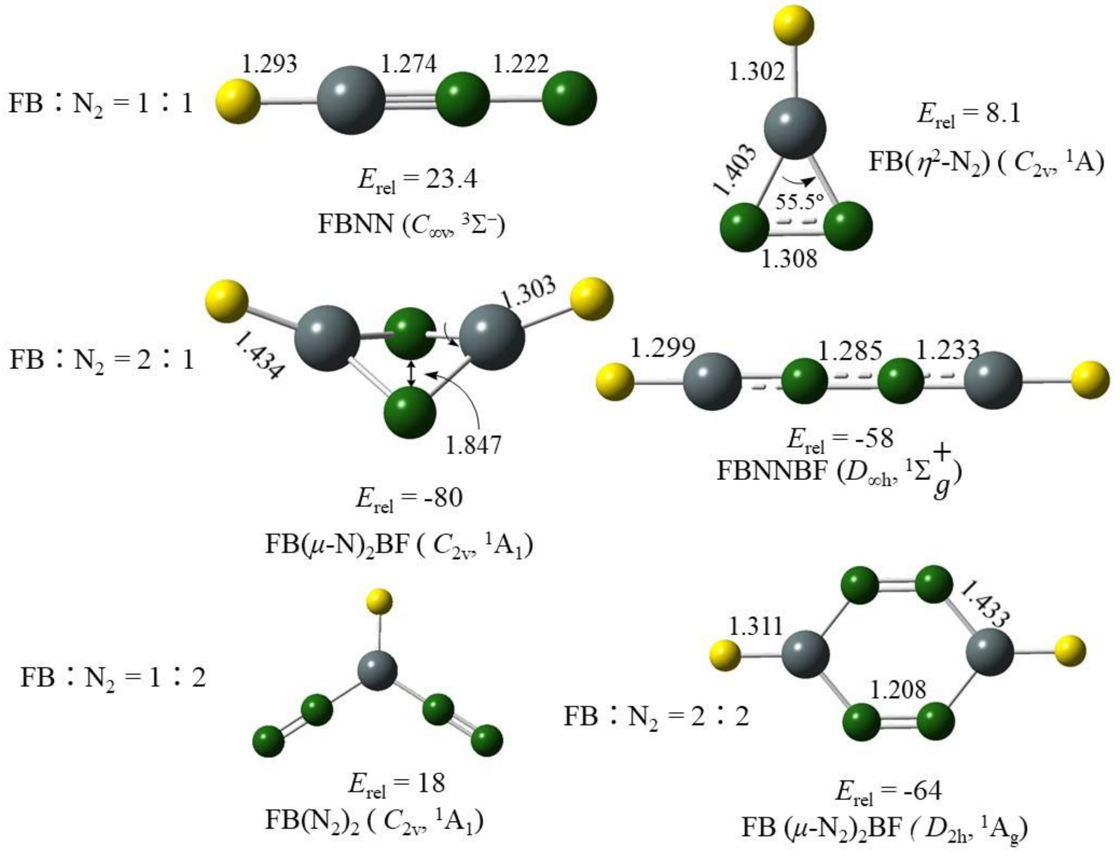

Figure S19. Oligomers of BF and N_2 calculated at B3LYP/6-311++G(3df, 3pd) level. Their relative energies are compared to the isolated reactants FB and N_2 in their respective ground electronic states.

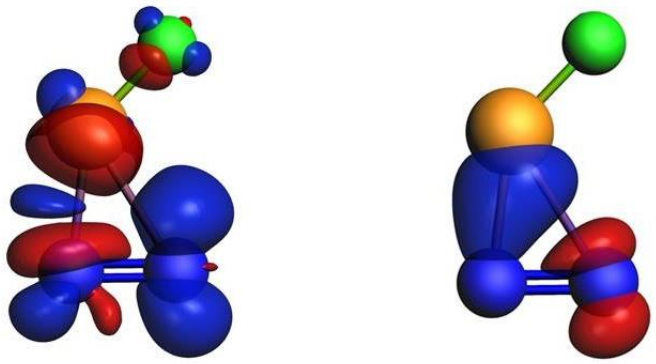

Figure S20. Plot of the deformation densities Δρ of the BF→N_2 donation and N_2→BF back-donation in TS6 (Figure S5) with the associated interaction energy ΔE_{orb} and charge eigenvalues $|\nu_n|$(in e). The the colour coding of the deformation densities $\Delta\rho(1)$ and $\Delta\rho(2)$ gives the charge flow from the red to the blue areas. These plots are obtained from an energy decomposition analysis (EDA) combined with the NOCV (natural orbitals for chemical valence) method [a)] showing the two most important pairs of interacting orbitals in the transition state TS6 (see Figure S5). While $\Delta E_{orb}(1)$ reveals σ donation from the FB lone pair mainly into the π* MO of N_2, but also in the newly formed B−N bond to the nearest N atom, the weaker $\Delta E_{orb}(2)$ interactions shows π donation from the N_2 fragment into the newly formed B−N sigma bonds. Note that both, the σ lone pair and the *in-plane* π* MO of FB are involved in the B−N bonds of the product, and that σ donation is the dominant contribution in TS6.

[a)]: L. Zhao, M. V. Hopffgarten, D. M. Andrada, G. Frenking, *Wiley Interdisciplinary Reviews: Computational Molecular Science.* **2018**, 8 (3), e1345.

Part 3: Supporting Tables

Table S1. Experimental frequencies [cm^{-1}] of binary boron nitrides observed in this work.[a)]

	$^{14}N^{11}B$	$^{14}N^{10}B$	$^{15}N^{11}B$	$^{15}N^{10}B$
NNBN	2126.2, 1806.7, 750.1	2126.4, 1864.2, 756.4	2054.5, 1785.9, 730.1	2057.0, 1845.4, 735.2
NBN	1589.9	1644.3	1575.1	1630.0
BBNN	1933.0, 1930.7	1937.0, 1933.0, 1544.4	1866.8	1871.2, 1540.8
BNBN	1964.0,1959.4	2006	/	/

[a)]Assignments were made in accordance with the previous work of P. Hassanzadeh, L. Andrews. *J. Phys. Chem.* **1992,** *96*, 9177–9182.

Table S2. Experimental frequencies [cm^{-1}] of binary boron fluorides.[a)]

	This work (N_2 matrix)		Previous work (Ar matrix [a)]	
	^{11}B	^{10}B	^{11}B	^{10}B
BF	1370.6	1412.5	1377.0	1419.3
BF_2	1384.8	1432.8	1390.1	1440.9
BF_3	1443.7, 1438.9, 1433.0	1497.5, 1495.1, 1490.0	1460.2, 1442.3, 1421.2	1509.1, 1497.2, 1472.0

[a)] a) B. Xu, L. Li, Z. Pu, W. J. Yu, W. J. Li, X. F. Wang, *Inorg. Chem.* **2019**, *58*, 2363-2371
b) B. Xu, W. J. Li, Z. Pu, W. J. Yu, T. F. Huang, J. J. Cheng, X. F. Wang, *Phys. Chem. Chem. Phys.* **2019**, *21*, 25577-25583
c) B. Xu, W. J. Li, W. J. Yu, Z. Pu, Z. Y. Tan, J. J. Cheng, X. F. Wang, L. Andrews, *Inorg. Chem.* **2019**, *58*, 13418-13425.

Table S3. Observed and Calculated Fundamental Frequencies (> 500 cm^{-1}) of cyclic FB(μ-N)$_2$BF ($^1A_1$) [a)]

Approximate mode description and mode symmetry	B3LYP/ 6-311++G(3df,3pd)	CCSD(T)/ def2-TZVP	Obs.	B3LYP/ 6-311++G(3df,3pd)	CCSD(T)/ def2-TZVP	Obs.
	**F^{11}B(μ-^{14}N)$_2$^{11}BF**			**F^{10}B(μ-^{14}N)$_2$^{10}BF**		
BF-BF stretch, a$_1$	1647.2 (54)	1664.3 (68)	1612.8	1710.7 (62)	1716.1 (70)	1660.7
BF-BF stretch, b$_2$	1571.2 (855)	1587.8 (802)	1531.0	1630.1 (918)	1635.9(850.0)	1590.2
B(NN)B ring, b$_1$	1133.8 (92)	1150.3 (123)	1090.7	1166.1 (78)	1177.0 (120)	1123.8
BNN ring, a$_1$	922.3 (0)	910.8 (0.5)		925.4 (0)	913.5 (1)	
BNN ring, b$_2$	864.4 (0)	871.3 (0)		870.5 (0)	890.9 (0)	
B(NN)B ring, a$_2$	847.7 (0)	855.2 (0.4)		864.5 (0)	855.3 (0)	
B(NN)B wag, a$_1$	685.5 (52)	688.0 (45)		708.3 (55)	706.8 (47)	
NBN bend, a$_1$	522.0 (0)	516.7 (0.5)		532.9 (42)	533.1 (40)	
B(NN)B deform, b$_2$	511.0 (38)	515.2 (37)		525.6 (0)	519.1	
	**F^{11}B(μ-^{15}N)$_2$^{11}BF**			**F^{10}B(μ-^{15}N)$_2$^{10}BF**		
BF-BF stretch, a$_1$	1642.5 (54)	1648.2 (57)	hidden	1706.1 (52)	1712.1 (64)	1662.8
BF-BF stretch, b$_2$	1566.0 (848)	1572.0 (785)	1521.4	1625.1 (911)	1631.3 (844)	1569.6
B(NN)B ring, b$_1$	1118.1 (72)	1128.5 (110)	1077.0	1151.0 (78)	1161.8 (118)	1108.5
BNN ring, a$_1$	900.9 (0)	888.9 (1)		904.1 (1)	892.3 (2)	
BNN ring, b$_2$	848.9 (0)	854.2 (0)		858.4 (0)	879.0 (0)	
B(NN)B ring, a$_2$	834.8 (0)	839.6 (0)		849.0 (0)	839.7 (0)	
B(NN)B wag, a$_1$	680.0 (50)	679.2 (43)		702.9 (54)	702.4 (46)	
NBN bend, a$_1$	514.8 (0)	510.9 (36)		532.7 (42)	532.9 (40)	
B(NN)B deform, b$_2$	510.8 (38)	508.1 (1)		518.0 (0)	510.9 (1)	
	**F^{11}B(μ-^{14}N)$_2$^{10}BF**			**F^{11}B(μ-^{15}N)$_2$^{10}BF**		
BF-BF stretch, a$_1$	1689.5 (102)	1694.8 (88)	1635.8	1684.8 (95)	1690.7 (87)	/
BF-BF stretch, b$_2$	1590.2 (793)	1595.7 (733)	1558.6	1585.0 (786)	1591.1 (727)	1542.0
B(NN)B ring, b$_1$	1150.6 (75)	1161.4 (115)	1101.5	1135.3 (75)	1146.0 (113)	/
BNN ring, a$_1$	923.8 (0)	911.9 (1)		902.5 (0)	890.6 (1)	
BNN ring, b$_2$	864.5 (0)	878.1 (0)		849.0 (0)	865.7 (0)	
B(NN)B ring, a$_2$	858.4 (0)	855.3 (0)		845.8 (0)	839.7 (0)	
B(NN)B wag, a$_1$	697.7 (53)	696.0 (46)		692.3 (52)	691.6 (45)	
NBN bend, a$_1$	524.7 (7)	521.5 (36)		521.3 (37)	521.2 (38)	
B(NN)B deform, b$_2$	520.4 (32)	517.5 (3)		516.1 (3)	509.5 (1)	

[a)] Intensities (in km mol^{-1}) are given in parentheses.

Table S4. Observed and Calculated Fundamental Frequencies (cm^{-1}) of linear FBNNBF ($^1\Sigma_g^+$). a)

Approximate mode description and symmetry	B3LYP/6-311++G(3df,3pd)	CCSD(T)/def2-TZVP	Obs.	B3LYP/6-311++G(3df,3pd)	CCSD(T)/def2-TZVP	Obs.
	$F^{11}B^{14}N^{14}N^{11}BF$			$F^{10}B^{14}N^{14}N^{10}BF$		
BN stretch, Σ_g^+	2309.7 (0)	2325.0 (0)		2361.4 (0)	2367.6 (0)	
BN stretch, Σ_u^+	2140.3 (1149)	2148.4 (943)	2078.2	2212.0 (1252)	2207.0 (1012)	2147.0
FB-BF stretch, Σ_g^+	1418.5 (0)	1423.7 (0)		1444.6 (0)	1444.5 (0)	
FB-BF stretch, Σ_u^+	1010.5 (185)	1007.8 (177)	981.0	1013.6 (180)	1010.2 (1174)	983.1
NN stretch, Σ_g^+	563.6 (0)	556.2 (0)		567.6 (0)	559.5 (0)	
FBN wag, Π_u	389.9 (53)	383.4 (49)		404.1 (56)	394.7 (51)	
FBNNBF deform, Π_u	387.9 (51)	383.0 (49)		402.0 (54)	394.3 (51)	
	$F^{11}B^{15}N^{15}N^{11}BF$			$F^{10}B^{15}N^{15}N^{10}BF$		
BN stretch, Σ_g^+	2268.7 (0)	2275.6 (0)		2323.2 (0)	2329.2 (0)	
BN stretch, Σ_u^+	2119.8 (1153)	2115.1 (933)	hidden	2192.4 (1256)	2188.1 (1018)	2125.0
FB-BF stretch, Σ_g^+	1397.4 (0)	1397.7 (0)		1420.9 (0)	1420.9 (0)	
FB-BF stretch, Σ_u^+	996.9 (174)	993.5 (168)	969.4	999.4 (170)	995.8 (165)	970.4
NN stretch, Σ_g^+	562.7 (0)	554.6 (0)		566.7 (0)	558.6 (0)	
FBN bend, Π_u	388.0 (52)	379.1 (47)		402.2 (56)	393.0 (51)	
FBNNBF bend, Π_u	385.9 (50)	378.7 (47)		400.1 (54)	392.7 (51)	
	$F^{11}B^{14}N^{14}N^{10}BF$			$F^{11}B^{15}N^{15}N^{10}BF$		
BN stretch, Σ_g^+	2340.7 (46)	2346.6 (35)		2302.2 (62)	2308.0 (45)	
BN stretch, Σ_u^+	2171.2 (1154)	2166.2 (934)	2108.8	2150.0 (1143)	2145.8 (929)	2073.4
FB-BF stretch, Σ_g^+	1431.3 (0)	1431.4 (0)		1409.0 (0)	1409.1 (0)	
FB-BF stretch, Σ_u^+	1012.0 (182)	1008.7 (176)	/	998.2 (172)	994.6 (166)	/
NN stretch, Σ_g^+	567.6 (0)	557.5 (0)		564.7 (0)	556.6 (0)	
FBN bend, Π_u	399.3 (50)	389.6 (46)		397.5 (49)	388.1 (45)	
FBNNBF bend, Π_u	396.4 (50)	388.3 (50)		394.7 (49)	386.6 (48)	

a) Intensities (in km mol^{-1}) are given in parentheses.

Table S5. Observed and Calculated Fundamental Frequencies (cm^{-1}) of FB(η^2-N_2) a)

Approximate mode description and symmetry	B3LYP/6-311++G(3df,3pd)	CCSD(T)/def2-TZVP	Obs.	B3LYP/6-311++G(3df,3pd)	CCSD(T)/def2-TZVP	Obs.
	$F^{11}B(\eta^2\text{-}^{14}N^{14}N)$			$F^{10}B(\eta^2\text{-}^{14}N^{14}N)$		
FB stretch, a_1	1759.6 (318)	1753.1	1710.5	1821.7(352)	1817.0	1765.8
NN stretch, a_1	1319.3(45)	1271.2	1229.5	1324.2(39)	1274.4	1231.0
NBN stretch, b_1	1181.2(6)	1183.2		1206.5(7)	1209.0	
NBN stretch, a_1	788.6 (3)	784.0)		788.8(3)	784.3	
FBN bend, b_1	467.2 (72)	467.6		485.6(77)	486.0	
FBN bend, b_2	407.6 (18)	413.8		414.7(19)	420.9 (0)	
	$F^{11}B(\eta^2\text{-}^{15}N^{15}N)$			$F^{10}B(\eta^2\text{-}^{15}N^{15}N)$		
FB stretch, a_1	1748.2	1743.7	1700.1	1811.5	1808.3	hidden
NN stretch, a_1	1280.9	1233.0	1192.5	1284.6	1235.4	hidden
NBN stretch, b_1	1158.9	1161.0		1184.8	1187.5	
NBN stretch, a_1	786.2	771.5		776.6	771.9	
FBN bend, b_1	466.0	466.3		484.4	484.8	
FBN bend, b_2	401.4	407.5		408.1	414.2	

a) Intensities (in km mol^{-1}) are given in parentheses.

Table S6. Optimized Ground-State Structures of FBNNBF and FB(μ-N)$_2$BF Obtained at the CCSD(T)/def2-TZVP Level of Theory

FBNNBF CCSD(T)/def2-TZVP optimized structure

N	0.648059	0.000296	-0.000089
N	-0.648046	0.000310	-0.000108
B	-1.886281	-0.000113	-0.000054
B	1.886295	0.000148	0.000018
F	3.191740	-0.000445	0.000050
F	-3.191727	-0.000151	0.000118

FB(*μ*-N)$_2$BF CCSD(T)/def2-TZVP optimized structure

F	-0.000000	-2.172035	-0.470311
B	0.000000	-0.980088	0.063325
N	0.943145	0.000001	0.555235
N	-0.943144	0.000001	0.555235
F	-0.000000	2.172023	-0.470311
B	-0.000000	0.980096	0.063333

Table S7. Computed Fundamental Frequencies (cm^{-1}) of Cyclic FB(η^2-N$_2$) (^{1}A$_1$) and Linear FBNN ($^3\Sigma^-$).

FB(N$_2$)		FBNN	
Approximate mode description and symmetry	B3LYP/6-311++G(3df,3pd) $^{a)}$	Approximate mode description and symmetry	B3LYP/6-311++G(3df,3pd) $^{a)}$
F-B stretch, a$_1$	1759.6(318)	B-N stretch, Σ^+	1997.4(222)
N-N stretch, a$_1$	1319.3(45)	F-B stretch, Σ^+	1390.5(165)
NBN stretch, b$_1$	1181.2(6)	N-N stretch, Σ^+	814.6(18)
NBN stretch, a$_1$	788.5(3)	BNN bend, Π	279.5(1)
F-BN bend, b$_1$	467.2(71)	BNN bend, Π	279.5(1)
F-BN bend, b$_2$	407.6(18)	FBN bend, Π	138.0(10) ×2

$^{a)}$ Intensities (in km mol^{-1}) are given in parentheses.

Table S8. Observed and Calculated Fundamental Frequencies (cm^{-1}) of Linear FBBF ($D_{\infty h}$, $^3\Sigma^-$) and Bent FBBF(C_{2h}, ^{1}A$_g$).

Approximate mode description and symmetry	F^{11}B^{11}BF			F^{11}B^{10}BF			F^{10}B^{10}BF		
	Linear FBBF ($D_{\infty h}$, $^3\Sigma^-$) $^{a)}$	Bent FBBF (C_{2h}, ^{1}A$_g$) $^{a)}$	Obs.	Linear FBBF ($D_{\infty h}$, $^3\Sigma^-$) $^{a)}$	Bent FBBF (C_{2h}, ^{1}A$_g$) $^{a)}$	Obs.	Linear FBBF ($D_{\infty h}$, $^3\Sigma^-$) $^{a)}$	Bent FBBF (C_{2h}, ^{1}A$_g$) $^{a)}$	Obs.
BB stretch, s	1848.3 (0)	1455.9(0)		1890.4(3)	1486.0(28)		1929.1(0)	1507.0(0)	
FB stretch, as	1341.0(574)	1353.8(568)	1327.3	1360.1(589)	1369.4(557)	1348.5	1382.6(610)	1395.3(604)	1370.3
FB stretch, s	637.8(0)	496.3(0)		639.3(0)	502.1(0)		640.8(0)	509.1(0)	
FBBF wag	241.8 (3)	368.2(0)		245.7(3)	373.6(0)		249.3(3)	378.3(0)	
FBBF wag	241.8 (3)	272.9(29)		245.7(3)	277.0(30)		249.3(3)	281.5(31)	
FBBF tors	170.5 (0)	169.4(1)		174.1(0)	172.6(1)		176.2(0)	175.3(1)	

$^{a)}$ B3LYP/6-311++G(3df,3pd) Level; Intensities (in km mol^{-1}) are given in parentheses.

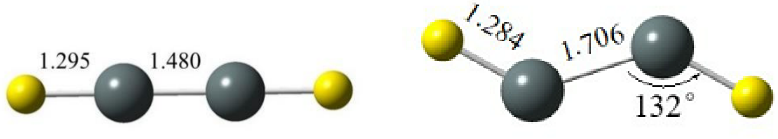

$D_{\infty h}$, $^3\Sigma^-$
−8.0 kcal mol^{-1}

C_{2h}, ^{1}A$_g$
0.0 kcal mol^{-1}

Table S9. Calculated Relative Energies for the Lowest Triplet and Singlet States of $(BF)_2(N_2)_n$ (n = 0, 1, 2, 3, 4) [a]

	n=0	n=1	n=2	n=3	n=4
^3A	0.00	0.00	0.00	0.00	0.00
^1A	+8.00	-10.93	-7.29	-6.14	-6.86
D	/	25.6	34.7	35.0	35.1

[a] Relative energies (kcal mol^{-1}) calculated at the B3LYP/6-311++g(3df,3pd) level. Computed dissociation energies *D* are given for the singlet species with n = 1 - 4 with respect to free F_2B_2 + n N_2; n = 0: linear FBBF ($D_{\infty h}$, $^3\Sigma^-$) and bent FBBF(C_{2h}, ^1A_g, Table S8).

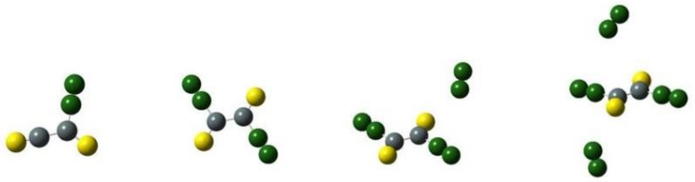

n=1 n=2 n=3 n=4

Singlet $(BF)_2(N_2)_n$ (n = 1,2,3,4)

Table S10. Computed Fundamental Frequencies of $FB{=}B(N_2)F$ (C_s, $^1A'$) and $F(N_2)B{=}B(N_2)F$ (C_{2h}, ^1A_g) and their Relative Energies with Respect to Free FBBF + n N_2 (n=1 or 2).

**FB=B(N₂)F**			**F(N₂)B=B(N₂)F**		
Approximate mode description and symmetry	B3LYP/6-311++G(3df,3pd) [a)]		Approximate mode description and symmetry	B3LYP/6-311++G(3df,3pd) [a)]	
N-N stretch, a′	2146.9(561)	2075.9(499)	N-N stretch, a_g	2221.7(0)	2147.1(0)
B-F stretch, a′	1625.0(683)	1623.9(699)	N-N stretch, b_u	2165.2(2207.5)	2092.1(2057)
B-F stretch, a′	1226.0(317)	1225.6(316)	B-F stretch, a_g	1394.7(0)	1394.1(0)
NBB stretch, a′	1005.0(27)	1000.6(25)	B-F stretch, b_u	1172.0(523)	1171.9(524)
NBB wag, a′	624.1(8)	617.4(8)	B-N stretch, a_g	1023.7(0)	1022.2(0)
BBN bend, a′	517.4(6)	511.8(6)	B-N stretch, b_u	759.5(239)	752.5(234)
NNB bend, a″	495.3(0)	484.1(0)	NNB bend, a_g	606.1(0)	598.2(0)
BBN bend, a′	462.7(7)	455.0(6)	NNB bend, b_g	547.3 (0)	544.7(0)
NNF wag, a″	359.4(2)	358.2(2)	NNB bend, b_u	534.9(7)	522.6(6)
NNF wag, a″	226.7(1)	224.8(1)	NNB bend, a_g	498.6(0)	487.0(0)
NNBF wag, a′	217.8(2)	215.3(2)	NN-B wag, a_g	446.6(0)	432.9(0)
NNBBF wag, a′	103.6(1)	102.2(1)	NN-B wag, a_g	351.6(0)	346.5(0)
			NN-B wag, b_g	320.8(0)	311.7(0)
			NBBN wag, a_u	258.2(4)	255.6(4)
			FBNN wag, b_u	245.5(3)	241.0(3)
			NNB wag, a_g	138.6(0)	136.0(0)
			NNBF tors, b_u	78.6(1)	77.1(1)
			NNBF tors, a_u	66.9(1)	65.6(1)

[a] Fundamental frequencies (in cm^{-1})) and intensities (in parentheses, in km mol^{-1}) of the most abundant ($^{11}B/^{14}N$, left column) and its ^{15}N (right column) isotopomer.

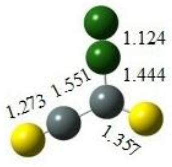

$FB{=}B(N_2)F$ (C_s, $^1A'$)
-25.6kcal mol^{-1}

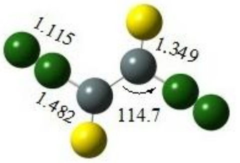

$F(N_2)B{=}B(N_2)F$ (C_{2h}, ^1A_g)
-34.7 kcal mol^{-1}
